# Predictors of dropout, time spent on the program and client satisfaction in an internet-based, telephone-assisted CBT anxiety program among elementary school children in a population-based sample

**DOI:** 10.1007/s00787-024-02486-8

**Published:** 2024-06-07

**Authors:** Katri Kaajalaakso, Terhi Luntamo, Tarja Korpilahti-Leino, Terja Ristkari, Susanna Hinkka-Yli-Salomäki, Andre Sourander

**Affiliations:** 1 Child psychiatry, University of Turku, Turku University Hospital, Turku, Finland; 2 INVEST Research Flagship, University of Turku, Turku, Finland

**Keywords:** Anxiety, Children, ICBT, Dropout

## Abstract

Increasing evidence has shown that childhood anxiety can be effectively treated by Internet-based cognitive behavioral therapy (ICBT). Being able to predict why participants decide to drop out of such programs enables scarce resources to be used appropriately. The aim of this study was to report dropout predictors for a population-based ICBT intervention aimed at children with anxiety, together with the time they and their parents spent on the program and client satisfaction rates. The study focused on 234 Finnish children aged 10–13 who received an ICBT intervention, with telephone support, for anxiety symptoms, as a part of a randomized control trial. Their parents also had access to Internet-based material and participated in the weekly telephone calls with the coach. Possible drop out factors were explored and these included various family demographics, child and parent psychopathology and therapeutic alliance. Just under a fourth (23.9%) of the children dropped out of the intervention. The risk was higher if the child did not fulfill the criteria for any anxiety diagnosis or reported a poorer therapeutic alliance. Family demographics and the COVID-19 pandemic did not increase the risk. The families spent an average of 127 min on the webpage each week and an average of 32 min on the phone calls. The overall satisfaction with the program was 87% for the children and 95% for the parents. Both the children and the parents found the telephone calls helpful. These findings are important in clinical practice when assessing a family’s eligibility for ICBT.

**Supplementary Information:**

The online version contains supplementary material available at 10.1007/s00787-024-02486-8.

## Introduction

Anxiety disorders are the most common psychiatric disorders among children [1] and they can be effectively treated by cognitive behavioral therapy (CBT) [2]. Untreated childhood anxiety tends to continue or increase the risk of other psychiatric disorders in adulthood [3–5]. However, most children with anxiety disorders do not receive evidence-based treatment [6, 7]. A number of randomized trials (RCTs) that provided children with Internet-based cognitive behavioral therapy (ICBT) have shown promising results [8–14]. This approach has potential when it comes to children participating in treatment, as they overcome many of the structural and logistic barriers associated with traditional CBT. These include long distances to the treatment centers and the impact that daytime meetings have on working parents.

Even if effective treatments are available, they cannot reach all the children who need them and some children and families drop out before they finish the intervention. The attrition rates for previous RCTs that have evaluated ICBT for childhood anxiety have ranged from 6 to 13% [8–13]. Wergeland et al. [15] have examined the risk factors for dropout in a clinic-based CBT program for childhood anxiety, and found out that low treatment credibility, reported by either child or parent, and parent self-rated internalizing symptoms were risk factors for discontinuing the CBT program. Fjermestad et al. [16] found that the child’s motivation and treatment credibility predicted child-rated therapeutic alliance in the early phase of face-to-face CBT, which suggests that the child’s attitudes prior to treatment may have a key role in the formation of the therapeutic alliance. It has also been reported that comorbid depression and ethnic minority status increase the risk for early dropout among youths who receive psychotherapy for anxiety [17]. The level of guidance provided by Internet-based interventions has also associated with the effectiveness of the program and the risk of participants dropping out [18]. Guided therapies provide the patient with the chance to contact a therapist or coach by email, a chat facility or telephone. This is provided in addition to the therapy content that the participant works through independently. A meta-analysis by Bennett et al. [19] compared guided and unguided self-help programs for children and adolescents with internal and external disorders. The authors reported that guided therapies provided significantly better efficacy.

Most studies on dropout rates during childhood anxiety psychotherapy programs have examined face-to-face CBT [15–17] and there has been a lack of research on the risk factors for dropouts during ICBT programs. In addition, ICBT trials have tended to target clinical samples [8–13].

The current study took place from August 2017 to August 2020. We recruited participants who screened positive for anxiety during routine medical examinations carried out by school health services. Children with subthreshold anxiety symptoms, with no diagnosed anxiety disorders, were also included. Participants completed a 10-week long program including weekly material and exercises in digital form, and weekly telephone calls with a coach. The weekly themes included psychoeducation about anxiety, techniques on how to ease anxiety, such as breathing techniques, as well as gradual exposure to the feared situations. This was the first study to assess the risk factors that led to children from a population-based sample to drop out of an ICBT program.

The first aim of this study was to report the completion rate for the ICBT intervention, and the factors associated with dropout. As many different factors have been associated with participation in ICBT in previous literature, such as low treatment credibility, parental internalizing symptoms, child depression and ethnic minority status, we have chosen a broad range of variables in the analysis, including both child and parent reported psychopathology measures as well as several demographic variables. The second aim was to report the time spent on the program by the children and their parents, namely the digital platform and telephone coaching. The third aim was to study how satisfied the participating families were with the treatment program. Our hypothesis, based on previous literature about remote parent training programs [20], was that children with more severe anxiety symptoms, and their families, would be more motivated to complete the program. On the other hand, we hypothesized that families with lower parental education levels would be less likely to complete the program, in line with previous studies [20, 21].

## Methods

The protocol for this RCT has previously been described in detail [22]. The study was an open two-parallel group RCT, stratified by sex, that compared a telephone-assisted ICBT initiative to an education control.

### Participants

The study population consisted of children aged 10–13 years who were in grades 4–6 at comprehensive schools in the Finnish cities of Turku, Tampere and Orivesi and the counties of North Karelia and Central Ostrobothnia. We also included children in grades 5–6 in the city of Espoo. The screening started in August 2017 in Turku and was extended to the other areas in 2018, and the last participants were randomized and completed the program during the spring 2020. The children were screened during their yearly school healthcare check-ups. If they screened positive for anxiety, we contacted their families to assess their eligibility for the RCT.

The inclusion criteria included scoring at least 22 points on the 41-item child-reported Screen for Child Anxiety Related Emotional Disorders (SCARED-C) questionnaire during the school health appointment. The exclusion criteria were assessed by the research team when we contacted the parents. These included no Internet access at home, insufficient Finnish or Swedish language skills to take part and visual or hearing impairments that hindered the use of the program. Children with an intellectual disability or autism spectrum disorder were excluded from the study. We also excluded children with suicidal intentions or a severe mental health disorder, those receiving ongoing psychotherapy and those whose medication for anxiety had changed during the last two months. As a result, 234 children were randomized to the intervention group, and 233 to the education control group. The analyses described in this article only focus on the intervention group. There was one participating parent for each child and this could be either their mother or father, or, in some cases, a step parent.

The mean age of the participating children was 11.5 years and 71.1% were girls. Of the 234 participating parents, 7.7% were 34 years of age or younger, 59.0% were 35–44 years of age and 33.3% were 45 years of age or older. Just under two thirds (63.7%) of the parents had a college or university degree, while 36.3% had a lower level of education. The prevalences of each anxiety disorder in the sample are shown in Supplementary Table 1.

**Table 1:** Overview of the treatment content

Theme	Content
Introduction	Presenting the intervention and tools
Theme 1. Learn to know anxiety	Psychoeducation
Theme 2. Deep breathing	Breathing techniques
Theme 3. Encouraging thinking	Learning how to change negative thoughts
Theme 4. Relaxation	Learning how to relax, positive parenting
Theme 5. Safe place	Learning how to use imaginary techniques
Theme 6. Anxiety ladders	Gradual exposure
Theme 7. Learn by practicing	Gradual exposure
Theme 8. Control your anxiety	Summary of the learned skills
Theme 9. Long-term plan	Maintenance plan, preventing setbacks
Booster call after 1 month	Follow up on skill practicing

### Measures

The school-based assessment for anxiety symptoms used five of the 41 items from the SCARED-C report [22]. Each item was scored from 0 to 2, with larger scores indicating higher levels of anxiety symptoms. The families were invited to participate in the study if the child had a total score of three or more points or scored the maximum of two points for any of the five items.

Before randomization, the parents answered questions about the family demographics, including the gender and age of their child, their own age, occupation and education level and the family’s structure and the languages the family spoke.

The parents provided details of any psychiatric symptoms that their child had by completing the Strengths and Difficulties Questionnaire (SDQ) [23]. The SDQ consists of 25 items, divided into five subscales of five questions, covering both positive and negative behaviors. The subscales are emotional symptoms, conduct problems, hyperactivity/inattention, peer relationship problems and prosocial behavior. Each item is rated on a scale of 0–2, with three possible answers: never, somewhat true and certainly true, respectively. The emotional symptoms and conduct problems subscales were considered most relevant for this study and were analyzed separately. When the parents had answered the SDQ element of the questionnaire they were asked how long the child’s difficulties had lasted and how severe the difficulties were. The SDQ has been validated in Finland and is considered reliable [24].

The full SCARED questionnaires were completed by the children (SCARED-C) and their participating parent (SCARED-P) once they had agreed to take part in the study. Both versions contain 41 items, consisting of five subscales. Four of these screen for specific anxiety disorders: panic disorder, generalized anxiety disorder, separation anxiety disorder and social anxiety disorder. There is also a subscale for school phobia, which is not classified as an anxiety disorder in the Diagnostic and Statistical Manual of Mental Disorders, Fourth Edition. The items are scored from 0 to 2, for not or hardly ever true, somewhat or sometimes true and very or often true. The maximum total score is 82. The SCARED questionnaires have been assessed in population-based samples in several countries. Four of the five subscales have proven highly reliable in cross-cultural settings, when measured by Cronbach’s alpha coefficient (alpha = 0.72–0.84). The exception is the school phobia subscale (alpha = 0.62) [25]. We have previously reported that the correlation between the child and parent reports in the Finnish SCARED is low, with children reporting higher anxiety levels than their parents [26].

Data on anxiety disorders were collected by asking the children and their parents to complete the Development and Well-Being Assessment (DAWBA) during a telephone interview. This includes both structured and open-ended questions and the answers are fed into a computer program that generates a summary sheet and predicts the most likely diagnoses. These results are then evaluated by a clinician, who confirms if the diagnostic criteria have been fulfilled [27]. The use of the DAWBA has been shown to increase the likelihood of diagnosing emotional disorders [28]. The content of the program was mainly designed to treat social anxiety and generalized anxiety disorders. As a result, we analyzed these diagnostic groups separately, in addition to the presence of any anxiety disorder in general.

The 21-item Depression Anxiety and Stress Scale (DASS-21) [29] was used to evaluate the parent’s personal stress, anxiety and depression. The items rate how well they apply to an individual, by using a four-point scale, ranging from zero for never to four for almost always. Cronbach’s alpha for the DASS-21 total has been quantified as alpha = 0.90, and the DASS-21 distinguishes well between depression, stress and anxiety [29].

After the children and their parents had completed the third iCBT module they filled in the 12-item Working Alliance Inventory - Short Revised (WAIS-SR) developed by Munder et al. [30]. When they had completed the whole iCBT program, they both filled in the eight-item Client Satisfaction Questionnaire (CSQ) from Attkisson et al. [31] online and also answered some questions about the general usability of the program at the same time.

The effect of the COVID-19 pandemic on the attrition rate was evaluated by using the date of the randomization as an indicator when the family entered the program. The 54 participants who had been randomized on 1 October 2019 or later were classified as potentially affected by COVID-19, as they completed at least a part of the program during the pandemic. Of these, 14 participants had been randomized on 1 February 2020 or later and had completed the whole program during the health crisis.

### Description of the intervention

The treatment comprised nine Internet-based modules, which included digital material for both the children and their parents, and weekly calls from their coach. The web application used Django, which is an open-source web framework written in Python, and all data gathered via the electronic platform was stored in PostgreSQL database.

During the calls, the coach spoke with the parent and then spoke with the child, when the parent was present. The most crucial parts of the Internet program included text, pictures, educational audio clips and animations. Each of the nine modules focused on specific themes (Table 1), including psychoeducation (theme 1), anxiety management skills (themes 2–5), gradual exposure (themes 6–8) and the maintenance plan (theme 9). The families also received a booster call approximately one month after they finished the treatment program.

### Statistical analyses

Logistic regression analysis was used to explore the risk of the families dropping out. The associations between completers and dropouts and family factors (family structure, parental age, and parental education level) as well as psychological measures (SCARED-C, SCARED-P, SDQ, DAWBA, parental DASS-21 and the child and parent versions of the WAI-SR) were performed in separate models. The statistical analyses were carried out using SAS statistical software, version 9.4 (SAS Institute Inc, Cary, North Carolina, USA). The data are reported using odds rations (OR) and 95% confidence intervals (CI).

The p-values are not corrected for the multiplicity given the exploratory nature of the study.

### Ethics

This study was performed in line with the principles of the Declaration of Helsinki. The study has been registered in ClinicalTrials. gov and approved by the research ethics board of the Hospital District of South West Finland (ETMK:67/1801/2017, approved on 20 June 2017). The children provided written, informed assent during the healthcare check-up and received verbal information on the study from the school nurse. The parents provided informed consent via an internet-based application that school personnel use to communicate with parents.

## Results

### Prevalence and predictors of dropout

**Fig. 1:**
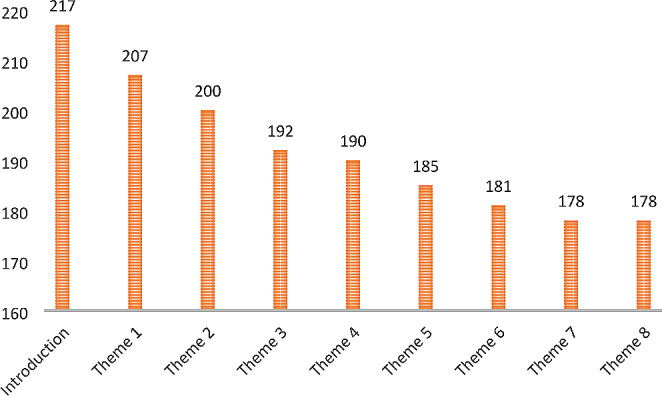
How many of the 234 children took part in each therapy module

More than three-quarters (76.1%) of the 234 children who were randomized to the intervention group completed all nine therapy modules, while the remaining 23.9% dropped out. Of the 56 who dropped out, 30.4% did not start the program after they were randomized, 30.4% dropped out after the introduction or during the first two weeks of the program and 39.3% dropped out during weeks 3–6 of the program. The numbers of participants who completed each therapy module are shown in Fig. 1.

Table 2 shows the associations between family factors and psychological measures and participation status. Only those children who completed all nine themes were classified as completing the program and those who completed 0–8 themes were classified as dropouts. Just under a fifth (19.7%) of those who fulfilled the diagnosis for any anxiety disorder dropped out, while 38.5% of those who did not fulfil the diagnostic criteria dropped out (OR 2.6, 95% CI 1.3-5.0, *p* = 0.006). These results indicate that fulfilling the diagnostic criteria for anxiety disorders was associated with completing the program. However, the majority (61.5%) of the children with subthreshold symptoms, namely elevated levels of anxiety that did not fulfill the diagnostic criteria, also completed the program.

**Table 2:** Family factors and psychological measures based on families who completed or dropped out of the program

Factors	Completed program (*n* = 178) *n* (%)	Dropped out (*n* = 56) *n* (%)	Unadjusted OR (95% CI)	*P*-value	Adjusted OR^a (^95% CI)	*P*-value
Age^h^						
10–11 years	99 (79.20%)	26 (20.80%)	0.68 (0.37–1.25)	0.22	0.68 (0.37–1.25)	0.21
12–13 years	78 (72.22%)	30 (27.78%)	1.0		1.0	
Child’s gender						
Female	129 (77.25%)	38 (22.75%)	0.80 (0.42–1.54)	0.51	0.81 (0.42–1.55)	0.52
Male	49 (73.13%)	18 (26.87%)	1.0		1.0	
Family structure						
Two biological parents	117 (79.59%)	30 (20.41%)	0.60 (0.33–1.11)	0.10	1.72 (0.93–3.19)	0.08
Non-nuclear family	61 (70.11%)	26 (29.89%)	1.0		1.0	
Parental age						
34 years or younger	13 (72.22%)	5 (27.78%)	1.45 (0.48–4.4)	0.75	1.62 (0.51–5.11)	0.57
35–44 years	109 (78.99%)	29 (21.01)	1.0		1.0	
45 years or older	56 (71.79%)	22 (28.21%)	1.48 (0.78–2.80)	0.59	1.37 (0.70–2.65)	0.86
Parental education level						
Secondary education or lower	60 (70.59%)	25 (29.41%)	1.0		1.0	
Higher education level	118 (79.19%)	31 (20.81%)	0.63 (0.34–1.16)	0.14	0.61 (0.33–1.13)	0.12
Any anxiety disorder^i^						
Yes	143 (80.34%)	35 (19.66%)	1.0		1.0	
**No diagnosis**	**32 (61.54%)**	**20 (38.46%)**	**2.55 (1.31–4.99)**	**0.006**	**2.82 (1.41–5.65)**	**0.004**
Generalized anxiety disorder diagnosis^j^						
Yes	90 (78.9%)	24 (21.1%))	1.0		1.0	
No	85 (73.9%)	30 (26.1%)	1.32 (0.72–2.44)	0.37	1.42 (0.76–2.68)	0.27
Social phobia diagnosis^j^						
Yes	57 (78.08%)	16 (21.92%)	1.0		1.0	
No	117 (75.00)%)	39 (25.00%)	1.19 (0.61–2.30)	0.61	1.25 (0.63–2.47)	0.52
Difficulties reported in SDQ						
Minor difficulties	123 (76.4%)	38 (23.60%)	1.0		1.0	
Moderate or severe difficulties	55 (75.34%)	18 (24.66%)	1.06 (0.56–2.02)	0.86	1.02 (0.53–1.95)	0.96
Length of difficulties						
Less than 6 months	28 (80.0%)	7 (20.0%)	1.0		1.0	
6 months or longer	150 (75.4%)	49 (24.6%)	1.3 (0.54–3.18)	0.56	1.19(0.49–2.93)	0.70
COVID-19						
Program completed before 30 September 2019	133 (73.9%)	47 (26.1%)	1.0		1.0	
Program fully or partly completed during the COVID-19 pandemic	45 (83.3%)	9 (16.7%)	0.57 (0.26–1.25)	0.16	0.64 (0.27–1.54)	0.32
*Continuous variables*	Mean (SD)	Mean (SD)				
SCARED-C^b, d^	34.6 (9.3)	33.0 (9.6)	0.98 (0.95–1.01)	0.25	0.98 (0.95–1.01)	0.24
SCARED-P^b, d^	24.4 (10.5)	23.1 (11.7)	0.99 (0.96–1.02)	0.44	0.99 (0.96–1.02)	0.43
SDQ^a, e^	11.3 (5.6)	12.0 (5.9)	1.02 (0.97–1.08)	0.45	1.02 (0.96–1.07)	0.60
Conduct problems score	5.5 (3.7)	5.9 (4.2)	1.03 (0.95–1.11)	0.51	1.02 (0.94–1.1)	0.62
Emotional score	5.80 (3.3)	6.1 (3.4)	1.02 (0.94–1.12)	0.60	1.02 (0.93–1.11)	0.74
**Child WAI-SR** ^**c, f,k**^	**46.6 (9.6)**	**34.7 (12.4)**	**0.9 (0.85–0.96)**	**0.001**	**0.91 (0.85–0.97)**	**0.003**
Parent WAI-SR^c, f,k^	51.2 (5.9)	47.9 (6.4)	0.92 (0.83–1.01)	0.09	0.92 (0.83–1.01)	0.09
Parental DASS-21^b, g^	16.5 (15.1)	14.7 (13.7)	0.99 (0.97–1.01)	0.44	0.99 (0.97–1.01)	0.48

^a^ Adjusted for age and sex

^b^ Higher scores indicate higher levels of symptoms

^c^ Higher scores indicate better therapeutic alliance

^d^ Screen for Child Anxiety Related Emotional disorders, C = child, D = parent

^e^ Strengths and Difficulties Questionnaire

^f^ Working Alliance Inventory, Short Revised

^g^ Depression, Anxiety, Stress Scale

^h^ 1 missing

^i^ 4 missing

^j^5 missing

^k^*N*=14. Child and parent WAI-SR were completed after module 3. Those children and parents (*N* = 42) who had dropped out earlier did not complete the questionnaire

Statistically significant values (*p* < 0.05) are shown in bold

Children who reported lower WAI-SR scores, indicating lower therapeutic alliance, had an increased risk of dropping out. Each point change was associated with an increased OR of 0.9 (95% CI 0.9–0.96, *p* = 0.001). The effect of the parental WAI-SR scores was close to significant (OR 0.9, 95% CI 0.8–1.01, *p* = 0.094). It is worth noting that the WAI-SR was only completed after week three and this means that scores were not obtained for participants who dropped out earlier.

The unadjusted analysis showed that a number of factors were not associated with the risk of dropping out of the program, including single parenthood (*p* = 0.10), parental age (*p* = 0.75 for parents aged 35 or younger, 0.59 for parents aged 45 or older) and education levels (*p* = 0.14). The factors also included the DASS-21 scores (*p* = 0.44), the DAWBA scores indicating general anxiety disorder (*p* = 0.37) or social phobia (*p* = 0.61), the SDQ total (*p* = 0.45), internalizing (*p* = 0.60) and externalizing sub scores (*p* = 0.51) and length (*p* = 0.56) and severity of difficulties (*p* = 0.86) and the SCARED child (*p* = 0.25) and parent scores (*p* = 0.44). There were no significant findings when all the variables were adjusted for the child’s age and gender (Table 2).

The COVID-19 pandemic was not associated with the risk of dropout. Just over a quarter (26.1%) of those who had been randomized by the end of September 2019 dropped out, compared with 16.7% who had been randomized later. The difference was not statistically significant (*p* = 0.16). Only 14 families were randomized in February 2020 or later and most of them completed the whole program during the start of the pandemic, as only two families (14.3%) dropped out.

**Fig. 2:**
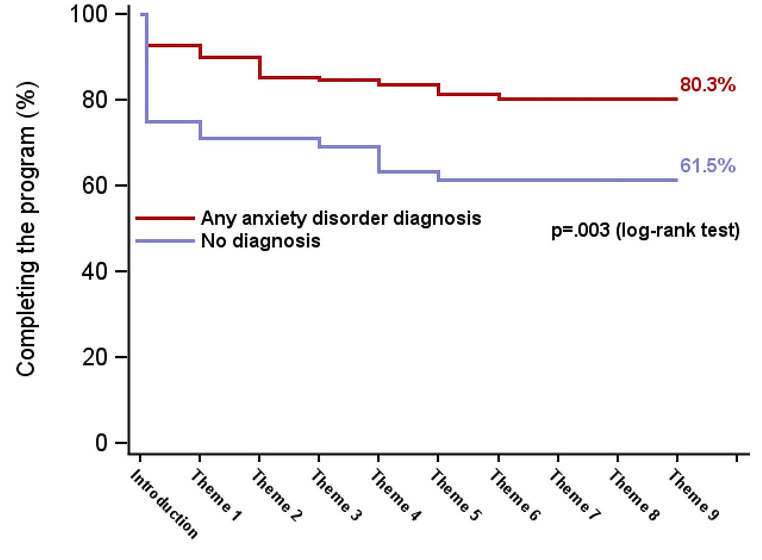
Percentages of participants who completed themes who completed themes by diagnosis status, based on the DAWBA interview

Figure 2 illustrates the numbers of participants who completed themes by diagnosis status, based on the DAWBA interview.

### Time spent on the program

The average time that the family spent exploring each theme on the website was 127 min (SD 73, range 38–420). This included the material aimed at both the parents and the children. The average length of the weekly telephone calls was 32 min (SD 8, range 4–58). The families spent an average of 2 h and 40 min a week engaging with the digital material and the telephone calls. The average total for those who completed the whole program was 25 h and 10 min (SD 11 h and 55 min, range 7 h and 40 min to 75 h and 40 min). It was not possible to measure the time that the families spent on the daily exercises and they are not included in these calculations. However, the program developers estimated that this might have been an average of 20 min a day.

### Client satisfaction

The program received very good ratings from the participating children and their parents when they completed the Client Satisfaction Questionnaire (Table 3). In general questions, most of the children (87.2%) and their parents (95.2%) reported that they were satisfied with the program in general. When they were asked about the usability of the program, 85.9% of the children thought that the skills training was helpful and 87.8% said that the weekly telephone calls with the coach were helpful. The percentages for the parents were 97.3% and 98.6%, respectively, indicating that parents rated the skills and telephone calls even more highly than their children.

**Table 3:** Percentages of participants who definitely agreed or agreed^a^ with the statements in the Client Satisfaction Questionnaire

	Children (*N* = 156)	Parents (*N* = 147)
The quality of service I / we received was good	84.6%	94.6%
I got the kind of service I wanted	88.4%	91.8%
The service has met my / our needs	87.8%	95.2%
I would recommend this program to a friend if s/he needed similar help	78.8%	93.9%
I am satisfied with the amount of help I/we received	91.0%	96.6%
The program made me / my child feel better	82.0%	89.8%
In an overall, general sense, I am satisfied with the program	87.2%	95.2%

^a^The other alternatives were neutral or no opinion, I disagree and I definitely disagree

## Discussion

This study produced three key findings. First, 23.9% of the children dropped out of this 10-week long, telephone-assisted iCBT program. Second, the risk of dropout was increased if the child did not fulfil the diagnostic criteria of any anxiety disorder diagnosis and they reported lower therapeutic alliance. Third, the program received excellent satisfaction ratings from both the parents and children. The COVID-19 pandemic, and the restrictions related to it, did not appear to affect drop-out rates.

An RCT by March et al. [8] reported a dropout rate of 20.5%, which was close to our result, while other studies have reported lower rates, varying from 6 to 13% [10–13]. It is important that any comparisons with other RCTs take into account that we used a population-based sample and used a strict definition of completion, which was only those children who had completed all nine therapy modules. Many of the previous RCTs stated that a significant number of participants failed to complete all the therapy modules, even though they may have participated in the telephone calls, and therefore they did not classify them as dropouts.

We found that 19.7% of the children with anxiety disorder diagnoses dropped out of our program, while 38.5% of those with subthreshold symptoms, who did not fulfil the diagnostic criteria, dropped out. This supports our hypothesis that the children with more severe anxiety are more likely to complete the program. However, it should be noted that even 61.5% of those with subthreshold symptoms completed the program. Those who fulfilled the diagnostic criteria were more likely to be more severely affected by anxiety symptoms and may have had higher motivation to complete the whole program. This indicates that the digital intervention, with telephone coaching, is useful in the treatment of more severe anxiety.

On the other hand, it must be noted that a majority of parents (161, 69%) reported in the SDQ that their child only had minor difficulties, indicating that the children in the sample were relatively high-functioning from a parent’s point of view. This can also indicate that the parents were not aware of their child’s anxiety at baseline. On the other hand, 77% of the participating children met the diagnostic criteria of an anxiety disorder in clinical evaluation – this is contradictory to the parental report that only a minority of children had severe difficulties, and highlights the importance of the child self-report and clinical evaluation when it comes to anxiety severity assessment.

A significant predictor of dropout was the child reporting lower therapeutic alliance, and the parent-reported therapeutic alliance was close to significant (*p* < 0.01). To our knowledge, there have been a lack of studies that have included a WAI-SR from children aged 10–13. Most of the previous studies that have examined therapeutic alliances in childhood psychotherapies have only measured these from the parental point of view [32, 33]. However, this finding, and the feedback ratings from the children and their parents, both supported the assumption that the telephone coaching would play an important role in the program. The satisfaction questionnaire showed that 87.8% of the children and 98.6% of the parents rated the telephone calls helpful. Previous research has shown a small, but significant, association between a good child-therapist alliance and improved therapy outcomes [34]. According to a meta-analyses by Bennett et al. [19], guided Internet-based therapies are more effective than unguided therapies for children and adolescents. However, there is limited evidence about how effective unguided ICBT is for children with anxiety. Based on these findings, we can assume that combining a good alliance with guidance has the potential to increase a child’s involvement and improve their ICBT results.

A positive, and somewhat unexpected finding, contradictory to our hypotheses, was that the family demographics in our study had no significant associations with the risk of dropout. This indicates that even families with less resources, such as single parents and those with lower education levels, were able to complete the program. Other studies have reported that lower maternal education level was associated with nonparticipation in an Internet-based parent training program for four-year-old children with conduct problems [20]. On the other hand, it must be noted that the educational level among the parents in our sample was higher than average: 69% of parents in this sample had a college or university degree, while the corresponding number for 40–44-year-olds in Finnish population on average was 47% in 2019 [35]. The majority of the participating families in our sample were recruited from urban areas and this is the most likely explanation for the higher educational level in our sample, as the educated population in Finland tends to concentrate in cities [35]. It is also possible that highly educated parents are more likely to participate scientific trials in general.

In addition, lower socioeconomic status has been reported to increase nonparticipation in parent training programs in general [21]. The present study showed that parental depression, anxiety and stress were not associated with the risk of dropout. These contradicted Wergeland et al. [15], who found that children of parents who self-reported internalizing symptoms were more likely to drop out of a clinic-based CBT for child anxiety. It is possible that it takes less time and energy to participate an ICBT program than CBT on a clinic and therefore the dropout rates may not be associated with parental factors. It is also noteworthy that during our program the parents received personal material and had telephone calls with a coach. Although the program was aimed at the children, parents with anxiety may have learned some skills to relieve their own personal symptoms.

A few limitations should be taken into account when interpreting these results. First, information about the therapeutical alliance was collected after the third weekly module and the WAI-SR results of the children and parents who dropped out earlier were not available. There were 192 children who filled in the WAI-SR after module 3, and only 14 of these dropped out. This means that of the 56 children who dropped out, only 25% had completed WAI-SR. This limits the conclusions that can be drawn from the predictive effect of treatment alliance. Second, the Client Satisfaction Questionnaire was only filled in by families who completed the intervention. Third, the findings related to the effects of the COVID-19 pandemic should be interpreted with caution because of the low number of participants in the analysis. It must also be noted that even though the number of participants was larger than in the previous ICBT RCT studies, it was still quite small, and the numbers of participants in different subgroups were especially small. For instance, the proportion of children without an anxiety disorder diagnoses was small and thus, the findings may not be fully generalizable to subclinical populations. Additionally, the sample was regional, i.e. restricted to different parts of Finland, and homogenic, as we excluded children whose Finnish or Swedish skills were insufficient. These issues restrict the generalization of the findings.

Only a small number of participants completed the whole program during the pandemic, but it is interesting that the COVID-19 pandemic did not increase the dropout rate. This might indicate that it is important to develop remote interventions for child and adolescent mental health that can be used during crises.

## Conclusion

In terms of high satisfaction and low attrition rate, this study supports the use of ICBT to treat children with anxiety following population-based screening by school health services. However, the dropout rate was approximately two-fold higher among children with subthreshold anxiety symptoms than those diagnosed with anxiety disorders. This may indicate that the program was too intensive for some children with milder symptoms. Children seemed to be more likely to continue their involvement with the program if they rated the therapeutic alliance highly, which should be considered in clinical practise. The children and their families were highly satisfied with the overall program and elements such as the telephone calls. These findings are important when planning population based ICBT interventions for children with anxiety.

## Electronic supplementary material

Below is the link to the electronic supplementary material.

Supplementary Material 1

## Data Availability

No datasets were generated or analysed during the current study.
