# Correction: Predictors of dropout, time spent on the program and client satisfaction in an internet-based, telephone-assisted CBT anxiety program among elementary school children in a population-based sample

**DOI:** 10.1007/s00787-024-02580-x

**Published:** 2024-10-12

**Authors:** Katri Kaajalaakso, Terhi Luntamo, Tarja Korpilahti-Leino, Terja Ristkari, Susanna Hinkka-Yli-Salomäki, Andre Sourander

**Affiliations:** 1 Child psychiatry, University of Turku, Turku University Hospital, Turku, Finland; 2 INVEST Research Flagship, University of Turku, Turku, Finland

**Correction: European Child & Adolescent Psychiatry**

10.1007/s00787-024-02486-8.

In this article, in the Abstract Section author would like to correct the text “Just under a fifth (23.9%)” it should be corrected as “Just under a fourth (23.9%)”.

This error has been updated in the original publication.

