# Supplementary material for: Predictors of dropout, time spent on the program and client satisfaction in an internet-based, telephone-assisted CBT anxiety program among elementary school children in a population-based sample
Source: Eur Child Adolesc Psychiatry. 2024 Jun 7;34(1):249–58. doi: 10.1007/s00787-024-02486-8 (PMC11805840; doi:10.1007/s00787-024-02486-8)
Supplement: Supplementary file 1 — Supplementary Material 1 [file 787_2024_2486_MOESM1_ESM.docx]

**Supplementary Table 1.** Percentages of participants who met the diagnostic criteria of each anxiety disorder based on the DAWBA interview. N=234. ^a^ 4 missing, ^b^ 5 missing.

| Diagnosis | Yes | No |
| --- | --- | --- |
| Separation anxiety disorder^a^ | 77 (32.9 %) | 153 (67.1 %) |
| Social phobia^b^ | 73 (31.2 %) | 156 (68.8 %) |
| Specific phobia^a^ | 125 (53.4 %) | 105 (46.6 %) |
| Panic disorder^a^ | 22 (9.4 %) | 208 (90.6 %) |
| Generalized anxiety disorder^b^ | 114 (48.7 %) | 115 (51.3 %) |
